# Proptosis is associated with thiol-disulfide in patients with Graves’ ophthalmopathy

**DOI:** 10.20945/2359-3997000000448

**Published:** 2022-03-23

**Authors:** Fettah Acibucu, Dilan Damla Öztürk, Cisem Kizildag, Muhammed Zubeyir Aslan, Erdinc Gulumsek, Merve Saracoglu Sumbul, Salim Neselioglu, Ozcan Erel, Suat Sen, Mehmet Bankir, Hilmi Erdem Sumbul

**Affiliations:** 1 University of Health Sciences – Adana Health Practice and Research Center Endocrinology Division Department of Internal Medicine Adana Turkey Department of Internal Medicine, Endocrinology Division, University of Health Sciences – Adana Health Practice and Research Center, Adana, Turkey; 2 University of Health Sciences – Adana Health Practice and Research Center Department of Internal Medicine Adana Turkey Department of Internal Medicine, University of Health Sciences – Adana Health Practice and Research Center, Adana, Turkey; 3 University of Health Sciences – Adana Health Practice and Research Center Department of Gastroenterology Adana Turkey Department of Gastroenterology, University of Health Sciences – Adana Health Practice and Research Center, Adana, Turkey; 4 Adana Provincial Health Directorate Department of Family Medicine Adana Turkey Department of Family Medicine, Adana Provincial Health Directorate, Adana, Turkey; 5 Ankara Yildirim Beyazit University Faculty of Medicine Department of Medical Biochemistry Ankara Turkey Ankara Yildirim Beyazit University Faculty of Medicine, Department of Medical Biochemistry, Ankara, Turkey

**Keywords:** Graves’ ophthalmopathy, proptosis, thiol-disulfide

## Abstract

**Objective:**

Graves’ ophthalmopathy (GO) is a vision-threatening finding observed in approximately half of Graves’ disease patients. The pathophysiology of GO is unclear, and one of the suspected factors is oxidative stress. In our study, we compared the relationship between proptosis and SH-SS in patients diagnosed with GO.

**Materials and methods:**

In this prospective study, 40 recently diagnosed Graves’ disease patients with proptosis, 40 recently diagnosed Graves’ disease patients without GO and 30 healthy individuals with similar demographic characteristics were included. Serum thiol-disulfide (SH-SS) measurements were performed. Eye examinations were performed by a single ophthalmologist to check for the presence of GO, and proptosis values were recorded with a Hertel exophthalmometer.

**Results:**

Total SH values were lower in the group with proptosis than in the other groups (p < 0.05). Total and native SH values were lower in patients without proptosis than in the control group (p < 0.05). Total SH, native SH and SS levels were independently associated with proptosis (p < 0.05). According to this analysis, it was found that increasing SS and decreasing total and native SH levels increased the probability of proptosis by 24.4%, 32.7% and 32.4%, respectively.

**Conclusion:**

A decrease in SH, which is a natural antioxidant that protects the body against oxidative stress, and an increase in SS are important signs of oxidative damage. Proptosis and SH-SS are closely related in GO. This may help us detect GO and proptosis in Graves’ patients. It can also assist in developing new options for preventing and treating GO.

## INTRODUCTION

Graves’ disease (GD) is an antibody-mediated autoimmune disease characterized by diffuse goiter and hyperthyroidism that can develop infiltrative ophthalmopathy ( 1 ). Proptosis, erythema in the periorbital tissues and conjunctiva, retraction and edema in the eyelids are signs of Graves’ ophthalmopathy (GO) ( 2 ). Thyroid stimulating hormone receptor antibody (TRAb) and its increase is important in the evaluation of disease severity, GO and proptosis ( 3 ).

Reactive oxygen species (ROS) are the primary molecules that cause oxidative damage when they increase above physiological levels ( 4 ). Thiol (SH) is an organic compound containing a sulfhydryl group that has a critical role in preventing the formation of any oxidative stress status in cells. With ROS, SH groups in the environment are oxidized and transformed into reversible disulfide (SS) bonds. This conversion is the earliest sign of radical-mediated protein oxidation ( 5 ). Dynamic SH-SS equilibrium has critical roles in antioxidant defense, detoxification, apoptosis, and the regulation of enzyme activities, transcription and cellular signal transduction mechanisms ( 6 ). The SS bond structures formed can be reduced back to the SH group, thus maintaining the SH-SS balance ( 7 ). SH-SS equilibrium measurements include native SH, dynamic SS, and total SH levels. While only one side of this reversible balance could have been measured since 1979, with the new method developed by Erel and Neselioglu, both levels of variables can be measured separately and cumulatively and they can be evaluated both individually and holistically ( 8 ).

Although TRAb and activated T cells play an important role in GO pathogenesis by activating retroorbital fibroblasts and adipocytes, the physiopathology of GO is still unclear ( 9 ). It has been concluded that free radicals are the primary cause of the symptoms seen in the pathogenesis during the later stages of GD ( 10 ). However, there are no data on the effects or the mechanism of oxidative stress on proptosis. In this study, we investigated whether proptosis and oxidative stress markers changed in GO patients and evaluated the relationship between these parameters. Oxidative stress markers may be an important predictor of proptosis development in GO patients. This study was conducted due to the lack of data on this subject in the literature.

## MATERIALS AND METHODS

This study was performed according to the tenets of the Declaration of Helsinki for research involving human subjects. Ethics Committee approval was obtained for the study. In the intervention study, using the G-Power program, when the effect size was taken as moderate and bidirectional and α = 0.05, power (1-β) = 0.80, the smallest sample size for each group was calculated as a minimum of 30 ( 11 ).

Our study was conducted prospectively with 40 Graves’ disease patients with recently diagnosed proptosis without comorbidities, 40 Graves’ disease patients without Graves’ ophthalmopathy and 30 healthy volunteers who were admitted between 03/02/2017 and 03/03/2018 to the Adana City Training and Research Hospital Internal Medicine Clinic, with the informed consent of the patients and volunteers. Patients with Graves’ disease were not using an antithyroid drug before participation. Patients were diagnosed based on their medical history, physical examination, imaging, and laboratory tests and those who provided consent to participate in the study were included. The exclusion criteria were smoking, alcohol use, drug use, hormone treatment, vitamin use, a systemic disease diagnosis (diabetes mellitus, hyperlipidemia and hypertension, etc.), malignancy, pregnancy, heart, lung, kidney, rheumatic and eye diseases. The same ophthalmologist examined all of the patients included in the study.

The EUGOGO was established in 1999. Europeans developed an assessment protocol for the evaluation of patients with GO based upon activity and severity parameters. Disease activity was evaluated based on the modified Clinical Activity Score (CAS). New patient and follow-up forms, together with the color atlas, may be downloaded from the EUGOGO website ( http://www.eugogo.eu/ ). Disease activity was assessed through rating the 10 items on the modified CAS. This CAS is based on four of the five well-known classical signs of inflammation (pain, redness, (warmth), swelling, and impaired function). For each of the 10 items present, one point is given. Each item has the same weight. The sum of these points is the CAS, which ranges from 0 to 10. Patients with active GO were not included in this study, and ophthalmopathy was considered to be active if the score was higher than or equal to 4/10. Both eyes were measured with a Hertel exophthalmometer. Over 20 mm was accepted as proptosis. These measurements were made by placing two ends of the exophthalmometer in the zygomaticofrontal sutures (lateral canthus), measuring the millimetric values in the mirror projection corresponding to the apex of both corneas, and taking the lateral orbital edge as the reference point. In patients whose right and left eye measurements were unequal, a higher value was recorded. In addition to the routine tests, native SH, SS and total SH levels of the patients included in the study were measured. For the EREL panel, blood samples were taken from the patients in a yellow-capped gel tube, and the serum part was separated by centrifugation at 2000 rpm for 10 minutes and stored at −80 degrees in Eppendorf tubes. The frozen tubes were sent to the Health Sciences University Ankara City Hospital Department of Medical Biochemistry. The samples were studied by Prof. Dr. Özcan EREL. The SS level was calculated with the formula (serum total SH – serum native SH)/2. Measurements were made with an Autocobas 501 autoanalyzer (Roche-Hitachi, Mannheim, Germany). Thyroid-stimulating hormone (TSH), free T3 (fT3) and free T4 (fT4) were measured by the chemiluminescent method (Beckman Coulter, DXI 800, Brea, CA, USA). A complete blood count was performed with a Sysmex XN 9000 brand device. Other biochemistry parameter measurements were studied with a Cobas C 701 brand biochemistry autoanalyzer (Roche, Germany).

### Statistical analyses

All analyses were performed using the SPSS 22.0 (Chicago, IL, USA) statistical software package. Variables were divided into two groups as categorical and continuous variables. Whether the distribution of continuous variables was normal was evaluated with the Kolmogorov–Smirnov test. Continuous variables are expressed as the mean ± SD. Categorical variables are given as numbers and percentages. Comparisons of the continuous variables were performed using one-way ANOVA or Kruskal-Wallis 1-way ANOVA according to the type of distribution. For normally distributed data, Scheffe and Games-Howell tests were used for multiple comparisons of groups regarding the homogeneity of variances. For nonnormally distributed data, the Bonferroni-adjusted Mann-Whitney U test was used for multiple comparisons. Statistical details between the groups are indicated in the tables. To independently identify patients with proptosis in GD, parameters with p < 0.01 and statistical significance in univariate analyses were included in the multivariate model, and multivariate logistic regression analysis was performed. A ROC curve analysis was performed to re-evaluate the independent markers for detecting patients with proptosis in GD and to determine the limit value of these markers. Parameters with an area under the curve (AUROC) > 0.70 were identified. Limit value determination was made for the best sensitivity and specificity of detecting patients with proptosis in GD among these parameters. Univariate correlation analysis was performed with Pearson's and Spearman's correlation methods to identify parameters related to eye measurements. Statistically significant parameters were included in a multivariate model, and linear regression analysis was performed with these parameters. Independent indicators affecting the eye measurements were identified. The mean of 6 eye measurements was taken to determine the proptosis status, and interclass correlation was used for reliability. Statistical significance was accepted as p < 0.05.

## RESULTS

### Comparison of the demographic, clinical and laboratory parameters of the study patients and the control group

Age, sex, body mass index, glucose, systolic blood pressure, diastolic blood pressure, aspartate aminotransferase, alanine aminotransferase, blood urea nitrogen, creatinine, low-density lipoprotein cholesterol, triglycerides, white blood cell, hemoglobin and platelet values were similar between the study patients and the control group ( Table 1 ). The interclass correlation result for all eye measurement values was found to be 94% reliable (p < 0,001). In the group with proptosis, the eye measurements and fT3 values were higher, and the total SH values were lower than in the other groups ( Table 1 ). The SS and fT4 values were higher and the native SH values were lower in patients with proptosis than in the control group ( Table 1 ). In patients without proptosis, the fT3 and fT4 values were higher, and the TSH, total SH and native SH values were lower than those in the control group ( Table 1 ). The TRAb value was higher in the group with proptosis than in the group without proptosis ( Table 1 ).

**Table 1:** Comparison of demographic, clinical and laboratory values of patients and control group

Variable	Control group n = 30	Patients with proptosis n = 40	Patients without proptosis n = 40	p
Age (year)	28.4 ± 2.28	28.3 ± 2.12	28.8 ± 1.93	0.540
Sex (female)	23 (76%)	27 (67%)	32 (80%)	0.425
Body mass index (kg/m^2^)	23.3 ± 1.30	23.3 ± 1.24	23.5 ± 1.27	0.780
Eye measurement (mm)	15.2 ± 0.80 β	15.0 ± 0.75 *	19.9 ± 1.97 β , *	**<0.001**
Systolic blood pressure (mmHg)	129.4 ± 7.14	132.5 ± 6.09	132.7 ± 6.07	0.070
Diastolic blood pressure (mmHg)	83.8 ± 5.03	84.5 ± 4.77	85.1 ± 5.12	0.561
Glucose (mg/dL)	97.8 ± 5.01	103.1 ± 13.8	101.2 ± 19.2	0.073
Aspartate aminotransferase (u/L)	20.7 ± 6.9	21.3 ± 4.7	23.6 ± 10.9	0.267
Alanine aminotransferase (u/L)	20.6 ± 14.7	22.9 ± 9.9	26.5 ± 21.7	0.309
Blood urea nitrogen (mg/dL)	25.5 ± 4.36	23.1 ± 4.21	24.4 ± 4.83	0.960
Creatinine (mg/dL)	0.47 ± 0.10	0.42 ± 0.11	0.44 ± 0.13	0.264
LDL cholesterol (mg/dL)	106.2 ± 17.7	99.8 ± 15.2	99.5 ± 30.9	0.286
Triglycerides (mg/dL)	133.3 ± 19.8	140.9 ± 11.9	136.3 ± 19.0	0.129
White blood cell (μL)	6.38 ± 1.52	7.17 ± 2.00	7.05 ± 1.73	0.160
Hemoglobin (g/dL)	13.8 ± 1.26	13.9 ± 1.71	13.9 ± 1.46	0.963
Platelet (K/mm^3^)	277.7 ± 25.9	283.7 ± 35.0	275.4 ± 37.8	0.140
fT3 (pg/mL)	3.48 ± 0.57 α , β	4.86 ± 1.36 α , *	6.77 ± 4.51 β , *	**<0.001**
fT4 (ng/dL)	0.82 ± 0.79 α , β	2.00 ± 0.64 α	2.33 ± 1.08 β	**<0.001**
TSH (uIU/dL)	1.93 ± 0.68 α , β	0.01 ± 0.00 α	0.01 ± 0.00 β	**<0.001**
TRAb (IU/l)		5.21 ± 1.10	48.5 ± 70.6	**<0.001**
Native SH (μmol/L)	382.6 ± 36.2 α , β	331.6 ± 13.6 α	319.3 ± 44.6 β	**<0.001**
Total SH (μmol/L)	415.3 ± 34.3 α , β	363.0 ± 10.2 α , *	333.4 ± 31.9 β , *	**<0.001**
SS (μmol/L)	16.2 ± 2.86 β	16.6 ± 4.49 *	20.5 ± 6.61 β	**0.002**

The values were shown as mean ± standard deviation or n (%). LDL: low density lipoprotein; fT3: free T3; fT4: free T4; TSH: thyroid stimulating hormone; TRAb: thyroid stimulating hormone receptor antibody; Total SH: Total Tiol; Native SH: Native Tiol; SS: Disulfide

α The significant association between the control group and group without GO (p < 0.05).

β The significant association between the control group and GO group (p < 0.05).

* The significant association between the group without GO and GO group (p < 0.05).

### Multivariate regression analysis to detect proptosis in patients with GD

In multivariate logistic regression analysis, it was found that total SH, native SH and SS levels independently predicted proptosis in patients with GD (p < 0.05, Table 2 ). According to the analysis in patients with GD, it was found that every 10 μmol/L decrease in total SH level, every 10 μmol/L decrease in native SH level and every 1 μmol/L increase in SS levels increased the possibility of proptosis by 24.4%, 32.7% and 32.4%, respectively ( Table 2 ).

**Table 2:** Multivariate regression analysis to detect proptosis in patients with GD

Proptosis	Odds Ratio	95% Confidence Interval	p
Total SH (10 μmol/L)	0.756	0.566-0.905	0.044
Native SH (10 μmol/L)	0.673	0.492-0.821	0.014
SS (1 μmol/L)	1.324	1.007-1.529	0.009

Abbreviations: Total SH: Total Tiol; Native SH: Native Tiol; SS: Disulfide.

### ROC analysis for detecting proptosis in patients with GD

**Figure 1:**
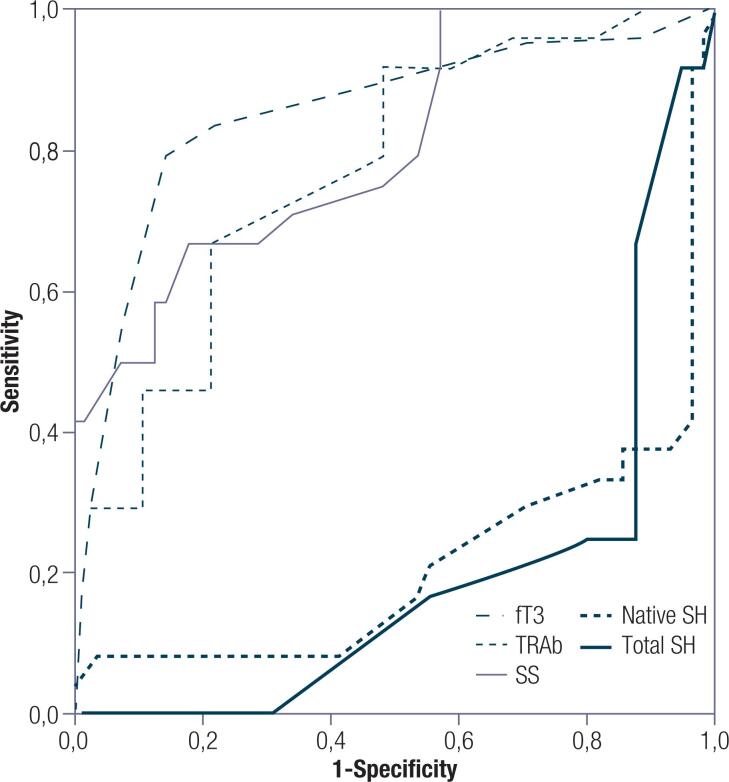
ROC analysis for detecting proptosis in patients with GD.

In the ROC analysis, the area under the curve was 0.772, 0.852, 0.804, 0.786 and 0.804 for the fT3, TRAb, total SH, native SH and SS values, respectively (p < 0.05, Table 3 and Figure 1 ). When the cutoff values for fT3, TRAb, total SH, native SH and SS were 4.81 pg/mL, 5.85 IU/l, 352.0 μmol/L, 326.4 μmol/L and 18.3 μmol/L, respectively, it identified proptosis in GD patients with 70.8% sensitivity and 69.6% specificity, 83.3% sensitivity and 78.6 specificity%, 75.0% sensitivity and 80.4% specificity, 70.8% sensitivity and 69.6% specificity, 70.8% sensitivity and 66.1% specificity, respectively ( Table 3 ).

**Table 3:** ROC analysis for detecting proptosis in patients with GD

Variable	AUROC Curve	P	Cut-off	Sensitivity	Specificity
fT3 (pg/mL)	0.772 (0.662-0.882)	0.010	4.81	70.8%	69.6%
TRAb (IU/l)	0.852 (0.751-0.953)	<0.001	5.85	83.3%	78.6%
Total SH (μmol/L)	0.196 (0.094-0.298)	<0.001	352.0	25.0%	19.6%
Native SH (μmol/L)	0.214 (0.088-0.340)	<0.001	326.4	29.2%	30.4%
SS (μmol/L)	0.804 (0.699-0.910)	<0.001	18.3	70.8%	66.1%

Abbreviations: fT3: free T3; TRAb: thyroid stimulating hormone receptor antibody; Total SH: Total Tiol; Native SH: Native Tiol; SS: Disulfide.

### Eye measurement related parameters

**Figure 2:**
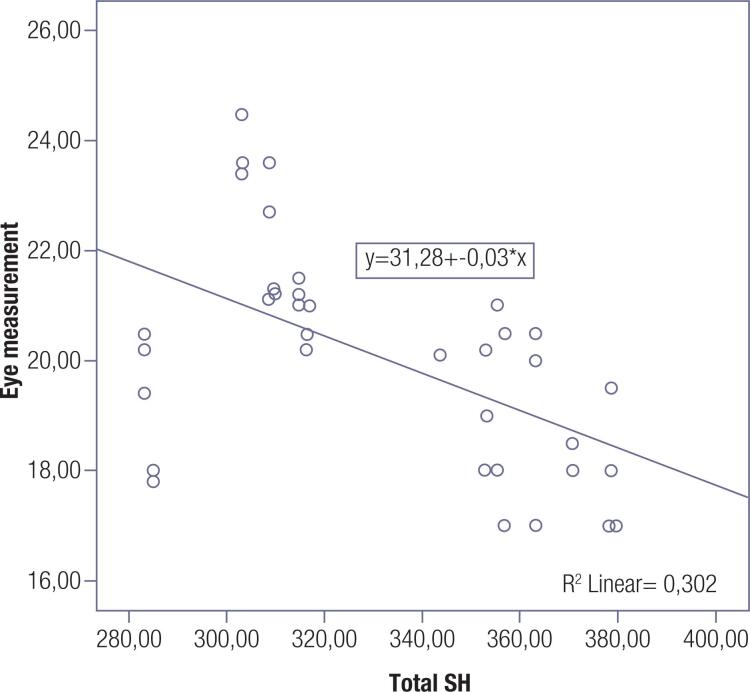
The relationship between eye measurement and total SH level.

Correlation analysis was performed between eye measurements and fT3, fT4, TRAb, native SH, total SH and SS ( Table 4 ). Linear regression analysis was performed using the parameters showing significant correlations with eye measurements ( Table 4 ). TRAb and total SH were found to be independently associated with the eye measurements ( Table 4 ). The relationship between the eye measurements and total SH level is shown in Figure 2 .

**Table 4:** The parameters associated with eye measurement and linear regression analysis for parameters significantly correlated with eye measurement in patients with proptosis

Variable	Univariate analyze		Multivariate analyze	
	p	r	p	β
fT3 (pg/mL)	**<0.001**	0.529	0.986	0.003
fT4 (ng/dL)	**0.001**	0.342	0.835	-0.022
TRAb (IU/l)	**<0.001**	0.696	0.041	0.424
Native SH (μmol/L)	0.076	-0.162	0.610	-0.044
Total SH (μmol/L)	**<0.001**	-0.678	**0.002**	-0.347
SS (μmol/L)	**<0.001**	0.422	0.285	0.106

fT3: free T3; fT4: free T4; TRAb: thyroid stimulating hormone receptor antibody; Total SH: Total Tiol; Native SH: Native Tiol; SS: Disulfide.

* ${\text{R}}_{\text{Adjusted}}^{2}$

## DISCUSSION

Oxidative stress occurs when the balance between ROS production and the antioxidant system is disrupted ( 4 ). One of the antioxidant mechanisms is SH-SS equilibrium. Evaluation of the SH-SS balance is critical to elucidating the effects of oxidative stress on the pathogenesis of diseases and evaluating responses to antioxidant therapies ( 12 - 13 ). Studies have shown that abnormal SH-SS balance levels are involved in the pathogenesis of various diseases, such as diabetes mellitus, cardiovascular diseases, Parkinson's disease, celiac disease and other inflammatory bowel diseases ( 14 - 17 ).

Mitochondrial energy production increases due to the acceleration of basal metabolism in GD; for this reason, oxygen consumption increases, and therefore, ROS increases ( 18 ). This increase is regulated by the antioxidant system. Tissue damage occurs if regulation is disrupted ( 19 ). Choi and cols. investigated oxidative stress markers, 8-hydroxy-2’-deoxyguanosine and malondialdehyde concentrations in tears of patients with GO and their relationship with the clinical activity score (CAS) and found that these values were higher in patients with active GO ( 20 ). This result proves that oxidant factors are involved in GO pathophysiology.

In this study, SH-SS levels were compared between the healthy control group and the Graves’ disease patients with and without proptosis. Total SH and native SH values were found to be lower in patients with proptosis, while SS values were higher. Our study added some new information to the literature about proptosis in GD. The first of these was that total SH, native SH and SS levels were independently associated with proptosis ( 21 ). Another important finding of our study is that fT3, TRAb, total SH, native SH and SS levels were different between the groups and could identify patients with a higher risk of proptosis.

In the SH-SS study conducted by Agan and cols. in patients with Graves’ disease, it was found that the total and native SH levels were lower and the SS values were higher than those of the control group ( 22 ). In another study conducted by Ademoğlu and cols. to determine oxidative stress in patients with Graves’ disease, total SH levels were found to be low in plasma ( 23 ). Similar results were found in our study as well. However, these studies did not evaluate proptosis.

A study conducted by Yuksel and cols. included smoking patients under treatment for GD who were not recently diagnosed and identified patients with active GO, and further found their mean SS levels were high and their native SH levels were low, similar to our study, and they found a correlation with CAS ( 24 ). However, they did not investigate proptosis. When we look at previous studies, our study is the first and only study in which eye measurements and SH-SS homeostasis were evaluated together in nonsmoking Graves’ disease patients. Therefore, our study may be more meaningful than the previous studies.

The most important limitations of our study are that our study was a single-centered, cross-sectional study that included a limited number of patients. More meaningful results could be obtained if eye measurements were evaluated by magnetic resonance or computed tomography and if a correlation analysis was performed between the severity of proptosis and antioxidant parameters. Another limitation was that it was not compared with other oxidant and antioxidant parameters, and the patients were not evaluated after treatment in our study. Histopathological evaluation could not be conducted in our study. Another limitation is that we had no data about the loss of weight or the estimated time of diagnosis.

In conclusion, as a result of oxidative stress developing in GD, the SH-SS balance is disrupted. In our study, it was found that proptosis values and SH-SS levels were closely related. Our study suggests that high levels of fT3 and TRAb cause changes in the SH-SS system. Measuring SH-SS levels can help to detect GO and proptosis in patients with Graves’ disease. It can also suggest new options for preventing and treating GO.
